# The genome sequence of the merveille du jour, *Griposia aprilina *(Linnaeus, 1758)

**DOI:** 10.12688/wellcomeopenres.18122.1

**Published:** 2022-10-04

**Authors:** Douglas Boyes, University of Oxford and Wytham Woods Genome Acquisition Lab, Darwin Tree of Life Barcoding collective, Wellcome Sanger Institute Tree of Life programme, Wellcome Sanger Institute Scientific Operations: DNA Pipelines collective, Tree of Life Core Informatics collective, David Lees, Darwin Tree of Life Consortium

**Affiliations:** 1 UK Centre for Ecology and Hydrology, Wallingford, Oxfordshire, UK; 2 Natural History Museum, London, UK

**Keywords:** Griposia aprilina, merveille du jour, genome sequence, chromosomal, Lepidoptera

## Abstract

We present a genome assembly from an individual *Griposia aprilina *(the merveille du jour; Arthropoda; Insecta; Lepidoptera; Noctuidae). The genome sequence is 720 megabases in span. The majority of the assembly (99.89%) is scaffolded into 32 chromosomal pseudomolecules with the W and Z sex chromosomes assembled. The complete mitochondrial genome was also assembled and is 15.4 kilobases in length.

## Species taxonomy

Eukaryota; Metazoa; Ecdysozoa; Arthropoda; Hexapoda; Insecta; Pterygota; Neoptera; Endopterygota; Lepidoptera; Glossata; Ditrysia; Noctuoidea; Noctuidae; Xyleninae; *Griposia*; *Griposia aprilina* (Linnaeus, 1758) (NCBI:txid1101106).

## Background

The merveille du jour, *Griposia aprilina* (Linnaeus, 1758), is a species of moth belonging to the Xylenini tribe of the Noctuidae family. The species is generally common but rarely abundant; it is widespread across Europe, observed as far east as the Urals, the Caucasus, and Asia Minor, as well as the British Isles (except for the extreme north and parts of Ireland), with a distribution increasing in the UK since 1970 ( Randle *et al.,* 2019).

*G. aprilina* is one of the most charismatic noctuids and adults are beautifully camouflaged with black, green, and white markings that mimic lichens on bark. The Linnean species name *aprilina* is thought to refer to the colour of opening buds, or spring ( Emmet, 1991). They prefer mature woodlands where larvae internally feed on flowers and leaves of oak trees ( *Quercus* spp.). Adults are on the wing between September and October and feed at night on ivy blooms and berries. They overwinter as eggs on branches or within bark of the host plant ( “Merveille Du Jour”, n.d.).

The moth may possibly be sister to the recently discovered *Griposia jahannamah* (belonging to BIN BOLD:ACJ6462 on BOLD) from Iran ( Fibiger *et al.,* 2008). It is very narrowly divergent in COI-5P to others of its BIN BOLD:AAC3647, including *G. wegneri*, *G. skyvai* and *G. bouveti* ( Huemer *et al.,* 2019) and also *G. pinkeri* of Greece and the Middle East, all of which are lichen-camouflaged. The genus does not yet seem to have been included in modern molecular phylogenetic works and it would be interesting to trace the evolution of colour pattern traits once the sister taxon of *Griposia* is known.

## Genome sequence report

**Figure 1.**
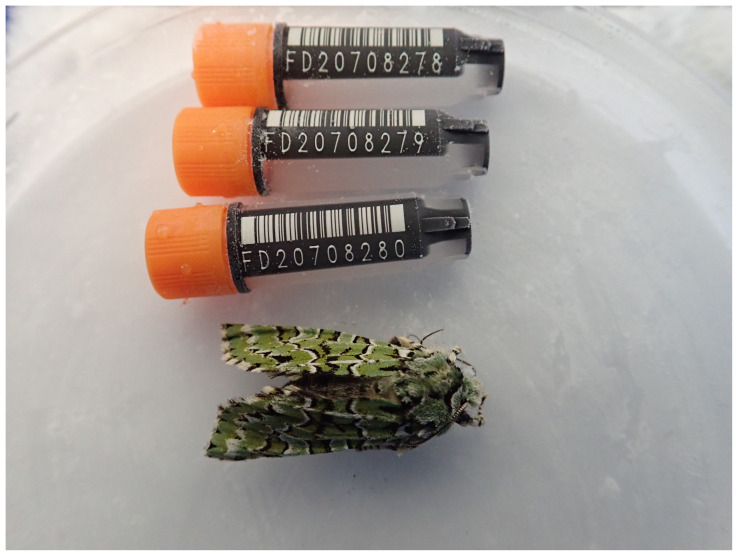
Image of the *Griposia aprilina* specimen taken prior to preservation and processing.

The genome was sequenced from a single female *G. aprilina* collected from Wytham Woods, Berkshire, UK ( Figure 1). A total of 31-fold coverage in Pacific Biosciences single-molecule HiFi long reads and 59-fold coverage in 10X Genomics read clouds were generated. Primary assembly contigs were scaffolded with chromosome conformation Hi-C data. Manual assembly curation corrected 26 missing/misjoins and removed four haplotypic duplications, reducing the assembly size by 0.17% and the scaffold number by 22.22%, and increasing the scaffold N50 by 4.16%.

**Figure 2.**
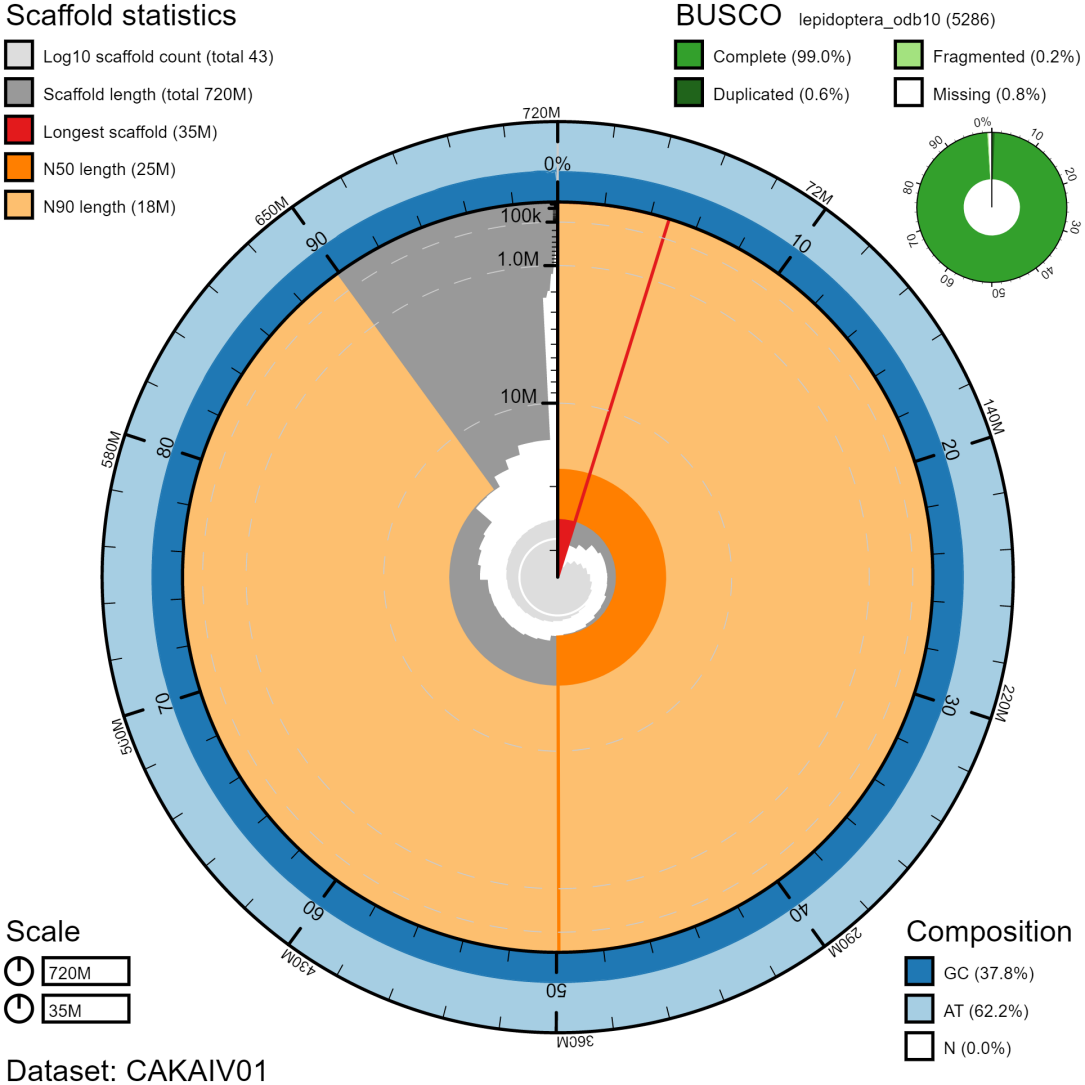
Genome assembly of *Griposia aprilina*, ilGriApri1.1: metrics. The BlobToolKit Snailplot shows N50 metrics and BUSCO gene completeness. The main plot is divided into 1,000 size-ordered bins around the circumference with each bin representing 0.1% of the 720,426,900 bp assembly. The distribution of chromosome lengths is shown in dark grey with the plot radius scaled to the longest chromosome present in the assembly (34,775,373 bp, shown in red). Orange and pale-orange arcs show the N50 and N90 chromosome lengths (24,562,086 and 17,579,852 bp), respectively. The pale grey spiral shows the cumulative chromosome count on a log scale with white scale lines showing successive orders of magnitude. The blue and pale-blue area around the outside of the plot shows the distribution of GC, AT and N percentages in the same bins as the inner plot. A summary of complete, fragmented, duplicated and missing BUSCO genes in the lepidoptera_odb10 set is shown in the top right. An interactive version of this figure is available at https://blobtoolkit.genomehubs.org/view/ilGriApri1.1/dataset/CAKAIV01.1/snail.

The final assembly has a total length of 720 Mb in 42 sequence scaffolds with a scaffold N50 of 24.6 Mb ( Table 1). The majority, 99.89%, of the assembly sequence was assigned to 32 chromosomal-level scaffolds, representing 30 autosomes (numbered by sequence length) and the W and Z sex chromosomes ( Figure 2– Figure 5; Table 2).

**Table 1.** Genome data for *Griposia aprilina*, ilGriApri1.1.

*Project accession data*	
Assembly identifier	ilGriApri1.1
Species	*Griposia aprilina*
Specimen	ilGriApri1 (genome assembly, Hi-C, RNA-Seq)
NCBI taxonomy ID	1101106
BioProject	PRJEB46317
BioSample ID	SAMEA8603200
Isolate information	Female. Thorax (genome assembly); head (Hi-C); abdomen (RNA-Seq)
*Raw data accessions*	
PacificBiosciences SEQUEL II	ERR6939240
10X Genomics Illumina	ERR6688515-ERR6688518
Hi-C Illumina	ERR6688401
PolyA RNA-Seq Illumina	ERR9435004
*Genome assembly*	
Assembly accession	GCA_916610205.1
*Accession of alternate haplotype*	GCA_916610245.1
Span (Mb)	720
Number of contigs	75
Contig N50 length (Mb)	18.6
Number of scaffolds	42
Scaffold N50 length (Mb)	24.6
Longest scaffold (Mb)	28.6
BUSCO * genome score	C:99.0%[S:98.4%,D:0.6%],F: 0.2%,M:0.8%,n:5,286

*BUSCO scores based on the lepidoptera_odb10 BUSCO set using v5.3.2. C= complete [S= single copy, D=duplicated], F=fragmented, M=missing, n=number of orthologues in comparison. A full set of BUSCO scores is available at https://blobtoolkit.genomehubs.org/view/ilGriApri1.1/dataset/CAKAIV01/busco.

**Figure 3.**
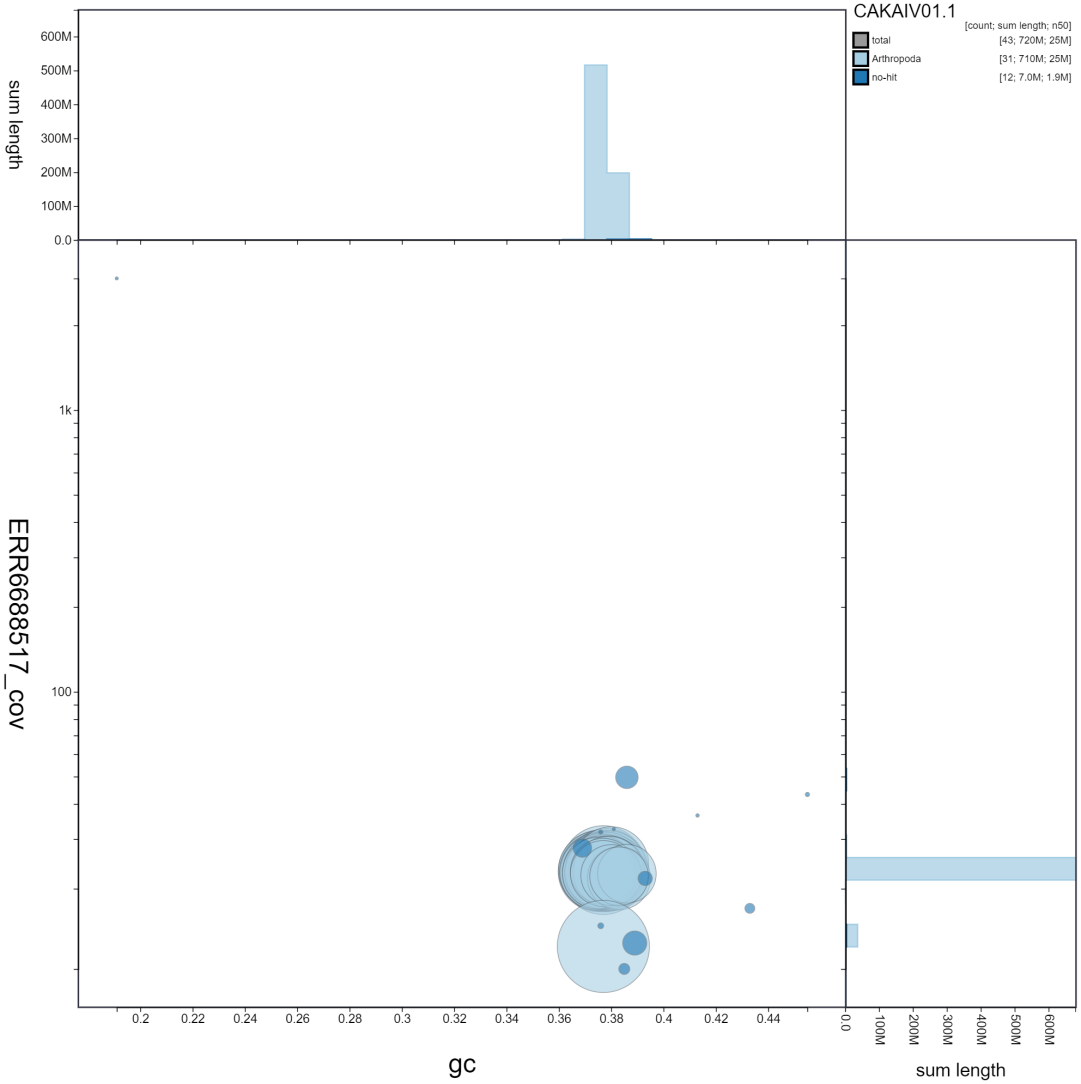
Genome assembly of *Griposia aprilina*, ilGriApri1.1: GC coverage. BlobToolKit GC-coverage plot. Scaffolds are coloured by phylum. Circles are sized in proportion to scaffold length. Histograms show the distribution of scaffold length sum along each axis. An interactive version of this figure is available at https://blobtoolkit.genomehubs.org/view/ilGriApri1.1/dataset/CAKAIV01.1/blob.

**Figure 4.**
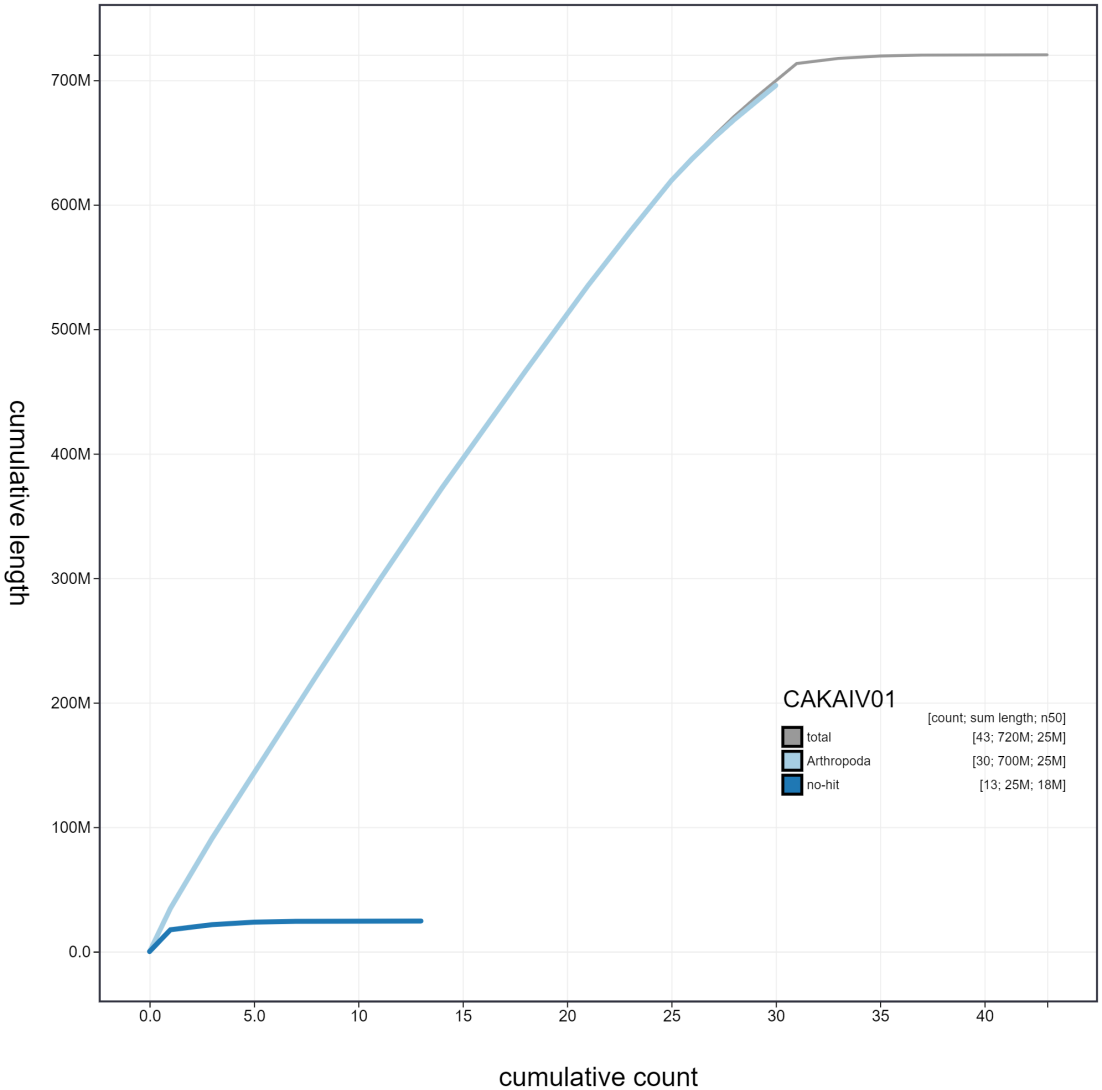
Genome assembly of *Griposia aprilina*, ilGriApri1.1: cumulative sequence. BlobToolKit cumulative sequence plot. The grey line shows cumulative length for all scaffolds. Coloured lines show cumulative lengths of scaffolds assigned to each phylum using the buscogenes taxrule. An interactive version of this figure is available at https://blobtoolkit.genomehubs.org/view/ilGriApri1.1/dataset/CAKAIV01.1/cumulative.

**Figure 5.**
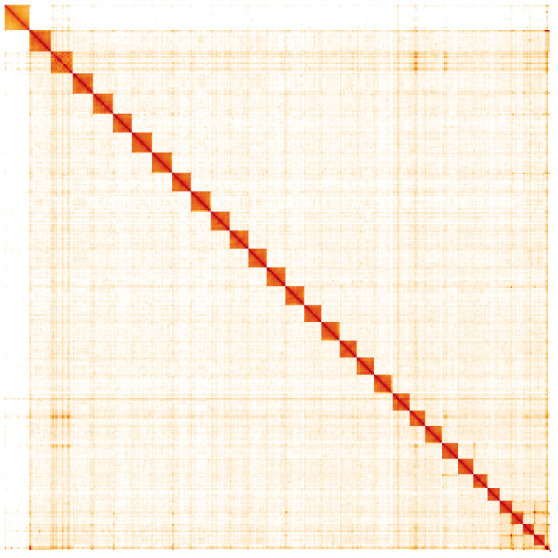
Genome assembly of *Griposia aprilina*, ilGriApri1.1: Hi-C contact map. Hi-C contact map of the ilGriApri1.1 assembly, visualised in HiGlass. Chromosomes are arranged in size order from left to right and top to bottom. The interactive Hi-C map can be viewed at https://genome-note-higlass.tol.sanger.ac.uk/l/?d=YUzUd2ygQNKfwuVpf4SRcA.

**Table 2.** Chromosomal pseudomolecules in the genome assembly of *Griposia aprilina*, ilGriApri1.1.

INSDC accession	Chromosome	Size (Mb)	GC%
OU744284.1	1	28.62	37.7
OU744285.1	2	27.79	37.7
OU744286.1	3	26.48	37.8
OU744287.1	4	26.07	37.6
OU744288.1	5	26.07	37.5
OU744289.1	6	26.02	37.7
OU744290.1	7	25.94	37.9
OU744291.1	8	25.62	37.5
OU744292.1	9	25.51	37.8
OU744293.1	10	25.19	37.6
OU744294.1	11	24.87	37.7
OU744295.1	12	24.79	37.9
OU744296.1	13	24.56	37.5
OU744297.1	14	23.77	37.5
OU744298.1	15	23.57	37.6
OU744299.1	16	23.31	37.8
OU744300.1	17	23.2	38
OU744301.1	18	23.06	37.8
OU744302.1	19	22.97	37.7
OU744303.1	20	22.86	38
OU744304.1	21	21.71	37.9
OU744305.1	22	21.26	37.6
OU744306.1	23	20.86	37.5
OU744307.1	24	20.55	37.9
OU744308.1	25	17.65	37.7
OU744309.1	26	17.58	37.7
OU744310.1	27	16.13	38.3
OU744311.1	28	14.95	38
OU744312.1	29	13.85	38.6
OU744313.1	30	13.84	38.3
OU744314.1	W	2.2	38.9
OU744283.1	Z	34.78	37.7
OU744315.1	MT	0.02	19.3
-	Unplaced	4.78	38.6

The assembly has a BUSCO v5.3.2 ( Manni *et al.,* 2021) completeness of 99.0% (single 98.4%, duplicated 0.6%) using the lepidoptera_odb10 reference set (n=5,286). While not fully phased, the assembly deposited is of one haplotype. Contigs corresponding to the second haplotype have also been deposited.

## Methods

### Sample acquisition and nucleic acid extraction

A single female *G. aprilina* specimen (ilGriApri1) was collected by using a light trap from Wytham Woods, Berkshire, UK (latitude 51.772, longitude -1.338) by Douglas Boyes (University of Oxford). The specimen was identified by Douglas Boyes and snap-frozen on dry ice.

DNA was extracted at the Tree of Life laboratory, Wellcome Sanger Institute. The ilGriApri1 sample was weighed and dissected on dry ice with tissue set aside for Hi-C sequencing. Thorax tissue was cryogenically disrupted to a fine powder using a Covaris cryoPREP Automated Dry Pulveriser, receiving multiple impacts. Fragment size analysis of 0.01-0.5 ng of DNA was then performed using an Agilent FemtoPulse. High molecular weight (HMW) DNA was extracted using the Qiagen MagAttract HMW DNA extraction kit. Low molecular weight DNA was removed from a 200-ng aliquot of extracted DNA using 0.8X AMpure XP purification kit prior to 10X Chromium sequencing; a minimum of 50 ng DNA was submitted for 10X sequencing. HMW DNA was sheared into an average fragment size between 12-20 kb in a Megaruptor 3 system with speed setting 30. Sheared DNA was purified by solid-phase reversible immobilisation using AMPure PB beads with a 1.8X ratio of beads to sample to remove the shorter fragments and concentrate the DNA sample. The concentration of the sheared and purified DNA was assessed using a Nanodrop spectrophotometer and Qubit Fluorometer and Qubit dsDNA High Sensitivity Assay kit. Fragment size distribution was evaluated by running the sample on the FemtoPulse system.

RNA was extracted from the abdomen tissue of ilGriApri1 in the Tree of Life Laboratory at the WSI using TRIzol, according to the manufacturer’s instructions. RNA was then eluted in 50 μl RNAse-free water and its concentration RNA assessed using a Nanodrop spectrophotometer and Qubit Fluorometer using the Qubit RNA Broad-Range (BR) Assay kit. Analysis of the integrity of the RNA was done using Agilent RNA 6000 Pico Kit and Eukaryotic Total RNA assay.

### Sequencing

Pacific Biosciences HiFi circular consensus and 10X Genomics Chromium read cloud sequencing libraries were constructed according to the manufacturers’ instructions. Sequencing was performed by the Scientific Operations core at the Wellcome Sanger Institute on Pacific Biosciences SEQUEL II (HiFi), Illumina NovaSeq 6000 (10X) and Illumina HiSeq 4000 (RNA-Seq) instruments. Hi-C data were generated in the Tree of Life laboratory from head tissue of ilGriApri1 using the Arima v2 kit and sequenced on a NovaSeq 6000 instrument.

### Genome assembly

Assembly was carried out with Hifiasm ( Cheng *et al.,* 2021); haplotypic duplication was identified and removed with purge_dups ( Guan *et al.,* 2020). One round of polishing was performed by aligning 10X Genomics read data to the assembly with longranger align, calling variants with freebayes ( Garrison & Marth, 2012). The assembly was then scaffolded with Hi-C data ( Rao *et al.,* 2014) using SALSA2 ( Ghurye *et al.,* 2019). The assembly was checked for contamination as described previously ( Howe *et al.,* 2021). Manual curation was performed using HiGlass ( Kerpedjiev *et al.,* 2018) and Pretext. The mitochondrial genome was assembled using MitoHiFi ( Uliano-Silva *et al.,* 2021), which performs annotation using MitoFinder ( Allio *et al.,* 2020). The genome was analysed and BUSCO scores generated within the BlobToolKit environment ( Challis *et al.,* 2020). Table 3 contains a list of all software tool versions used, where appropriate.

**Table 3.** Software tools used.

Software tool	Version	Source
Hifiasm	0.15.3	Cheng *et al.,* 2021
purge_dups	1.2.3	Guan *et al.,* 2020
SALSA2	2.2	Ghurye *et al.,* 2019
longranger align	2.2.2	https://support.10xgenomics.com/ genome-exome/software/pipelines/ latest/advanced/other-pipelines
freebayes	1.3.1-17-gaa2ace8	Garrison & Marth, 2012
MitoHiFi	2.0	Uliano-Silva *et al.,* 2021
HiGlass	1.11.6	Kerpedjiev *et al.,* 2018
PretextView	0.2.x	https://github.com/wtsi-hpag/ PretextView
BlobToolKit	3.2.6	Challis *et al.,* 2020

### Ethics/compliance issues

The materials that have contributed to this genome note have been supplied by a Darwin Tree of Life Partner. The submission of materials by a Darwin Tree of Life Partner is subject to the Darwin Tree of Life Project Sampling Code of Practice. By agreeing with and signing up to the Sampling Code of Practice, the Darwin Tree of Life Partner agrees they will meet the legal and ethical requirements and standards set out within this document in respect of all samples acquired for, and supplied to, the Darwin Tree of Life Project. Each transfer of samples is further undertaken according to a Research Collaboration Agreement or Material Transfer Agreement entered into by the Darwin Tree of Life Partner, Genome Research Limited (operating as the Wellcome Sanger Institute), and in some circumstances other Darwin Tree of Life collaborators.

## Data availability

European Nucleotide Archive: Griposia aprilina (merveille du jour). Accession number PRJEB46317; https://identifiers.org/ena.embl/PRJEB46317 ( Wellcome Sanger Institute, 2022)

The genome sequence is released openly for reuse. The *G. aprilina* genome sequencing initiative is part of the Darwin Tree of Life (DToL) project. All raw sequence data and the assembly have been deposited in INSDC databases. The genome will be annotated using the RNA-Seq data and presented through the Ensembl pipeline at the European Bioinformatics Institute. Raw data and assembly accession identifiers are reported in Table 1.
