# Supplementary material for: The genome sequence of the merveille du jour, Griposia aprilina (Linnaeus, 1758)
Source: Wellcome Open Res. 2022 Oct 4;7:247. [Version 1] doi: 10.12688/wellcomeopenres.18122.1 (PMC9971638; doi:10.12688/wellcomeopenres.18122.1)
Supplement: Peer review history

# Peer review: Reviewer response for version 1

**DOI:** 10.21956/wellcomeopenres.20093.r54732

**Published:** 2023-02-23

**Authors:** Simon H. Martin

**Affiliations:** 1 University of Edinburgh, Edinburgh, UK

This report describes the assembly of an excellent quality genome of the moth *Griposia aprilina*. I have no concerns about the protocol and resulting assembly.

 I found one issue with the report: Table 1 says the longest scaffold is 28.6 Mb, but I believe this is the longest autosome. It seems that the longest scaffold overall is the Z chromosome, at 35 Mb (as given in Figure 2 and caption).

Are sufficient details of methods and materials provided to allow replication by others?

Yes

Is the rationale for creating the dataset(s) clearly described?

Yes

Are the datasets clearly presented in a useable and accessible format?

Yes

Are the protocols appropriate and is the work technically sound?

Yes

Reviewer Expertise:

Evolutionary genomics

I confirm that I have read this submission and believe that I have an appropriate level of expertise to confirm that it is of an acceptable scientific standard.

# Peer review: Reviewer response for version 1

**DOI:** 10.21956/wellcomeopenres.20093.r54269

**Published:** 2023-02-06

**Authors:** Vladimir A. Lukhtanov

**Affiliations:** 1 Department of Karyosystematics, Zoological Institute of Russian Academy of Sciences, St. Petersburg, Russian Federation

The authors present a chromosome-scale genome assembly for the noctuid moth *Griposia aprilina. *The note provides brief information about the species, its genome, and the methods that were used to sequence and assemble the genome. This high-quality assembly will serve as a tool for further studies of the genus *Griposia, *which include several recently described closely related species with unclear taxonomic status. It is valuable that the female was studied and this made it possible to assemble not only the sex Z chromosome, but also the W chromosome, which turned out to be tiny (the smallest element in the set).

 I have no serious points for criticism, but I have the following minor suggestions. Since, once genome-wide data is obtained, the studied specimen becomes a kind of reference for the whole species, it would be extremely useful to have complete label data for this specimen. Such data is mainly in the article, with the exception of information about the date of collection and where the studied sample is stored. I understand that the butterfly's body was used for DNA extraction, however the wings remain which carry important information needed for identification, and the location of the institute/museum where the voucher is kept may be of value to future researchers.

 It also seems to me that it would be very useful to figure this voucher sample and to take and include in the article photographs of these wings (both forewings and hindwings, both upper side and underside), in addition to the Figure 1.

Are sufficient details of methods and materials provided to allow replication by others?

Yes

Is the rationale for creating the dataset(s) clearly described?

Yes

Are the datasets clearly presented in a useable and accessible format?

Yes

Are the protocols appropriate and is the work technically sound?

Yes

Reviewer Expertise:

Cytogenetics, Lepidoptera taxonomy

I confirm that I have read this submission and believe that I have an appropriate level of expertise to confirm that it is of an acceptable scientific standard.
